# Psychological stress induced bladder overactivity in female mice is associated with enhanced afferent nerve activity

**DOI:** 10.1038/s41598-021-97053-5

**Published:** 2021-09-01

**Authors:** Kylie A. Mills, Eliza G. West, Donna J. Sellers, Russ Chess-Williams, Catherine McDermott

**Affiliations:** Centre for Urology Research, Faculty of Health Sciences and Medicine, Bond University, Gold Coast, QLD 4229 Australia

**Keywords:** Neuroscience, Physiology, Urology

## Abstract

Psychological stress has been linked to the development and exacerbation of overactive bladder symptoms, as well as afferent sensitisation in other organ systems. Therefore, we aimed to investigate the effects of water avoidance stress on bladder afferent nerve activity in response to bladder filling and pharmaceutical stimulation with carbachol and ATP in mice. Adult female C57BL/6J mice were exposed to either water avoidance stress (WAS) for 1 h/day for 10 days or normal housing conditions. Voiding behaviour was measured before starting and 24-h after final stress exposure and then animals were euthanised to measure afferent nerve activity in association with bladder compliance, spontaneous phasic activity, contractile responses, as well as release of urothelial mediators. WAS caused increased urinary frequency without affecting urine production. The afferent nerve activity at low bladder pressures (4–7 mmHg), relevant to normal physiological filling, was significantly increased after stress. Both low and high threshold nerves demonstrated enhanced activity at physiological bladder pressures. Urothelial ATP and acetylcholine release and bladder compliance were unaffected by stress as was the detrusor response to ATP (1 mM) and carbachol (1 µM). WAS caused enhanced activity of individual afferent nerve fibres in response bladder distension. The enhanced activity was seen in both low and high threshold nerves suggesting that stressed animals may experience enhanced bladder filling sensations at lower bladder volumes as well as increased pain sensations, both potentially contributing to the increased urinary frequency seen after stress.

## Introduction

Psychological stressors can be defined as perceived threats to well-being that tax or exceed an individual’s capacity to cope with such threats^1^ and can come in many forms from chronic daily pressures of life to single severe events of violence or trauma. Stressors of various severities have been shown to affect health with the progression to disease being affected by the severity, frequency and persistence of the stressors as well as the individual’s genetics, constitution, learned coping skills and support resources^2^. There is a growing body of clinical evidence linking overactive bladder (OAB) and interstitial cystitis/bladder pain syndrome (IC/BPS) with psychological stress and stress disorders^3–10^ with both patient groups reporting significantly higher psychological stress levels than healthy controls^5^. Stress appears to have a role in both the development of bladder symptoms^3^ and in worsening existing symptoms^5,7^.

The storage and micturition reflexes of the bladder are controlled by information from the afferent nerves and changes in their activity result in bladder dysfunction such as OAB and IC/BPS^11,12^. Stress has been shown to affect activity of the afferent nerves in the gastrointestinal system leading to dysfunction such as dyspepsia and irritable bowel syndrome. One such study by Li et al.^13^, demonstrated that 8 weeks of mild stress in male mice resulted in enhanced mechanosensitivity of both the mucosal and tension-sensitive gastric vagal afferents. While rats exposed to water avoidance stress (WAS) had enhanced abdominal withdrawal reflexes in response to colonic distension compared to controls which suggests afferent hypersensitivity after stress^14^.

It has been postulated that the stress induced afferent sensitisation seen in the intestine may also occur in afferents arising in the bladder. Female rats exposed to WAS demonstrated voiding at lower bladder volumes compared to controls as well as enhanced responses to cold saline infusion suggesting a role for afferent nerves in the stress induced bladder overactivity^15^. To our knowledge the only study to directly record the activity of bladder afferent nerve fibres after stress was in young male mice in the context of social defeat stress related bladder disorders in children^16^. Specifically, 6 week-old male mice were placed individually in a cage with an aggressive retired breeder mouse and once aggressive behaviour was initiated a clear plastic barrier was inserted to physically separate them while maintaining visual and olfactory contact for a period of 1 h a day for 14 days. Cystometry in the young mice showed that intermittent social stress caused overactive bladder. Additionally, using an ex vivo bladder preparation where increasing bladder pressure stimulated afferent nerve activity, stressed bladders generated significantly more nerve impulses at each pressure increment compared to control bladders. However, stressed bladders also had increased compliance, requiring much larger fill volumes to reach each comparable pressure increment, which impacts the interpretation of the nerve activity data. The overactive phenotype observed by Mingin et al.^16^, also contrasts with reports by several other groups who observed urinary retention in male social defeat rodent models in both young^17^ and adult animals^18,19^. However, previous work by Mingin et al.^20^ demonstrated that in young male mice social defeat over 14 days had differing effects depending on the duration of the stress. Specifically, sensory exposure to an aggressor mouse for either 1 h or 24 h daily causing bladder overactivity or retention respectively indicating that continual stress is required in young mice to develop urinary retention.

Bladder disorders predominately affect adult women^21,22^ and our group have previously observed an overactive voiding phenotype in adult female mice following WAS^23^. Therefore, we aimed to use a WAS model in adult female mice to investigate the effects of psychological stress on bladder afferent nerve activity.

## Results

### WAS and overactive bladder symptoms

**Figure 1:**
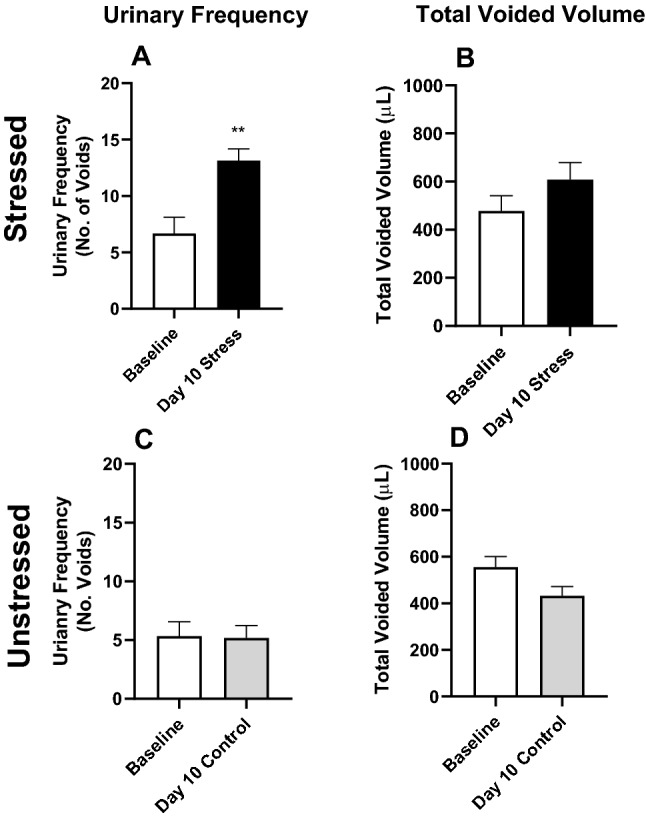
Urinary frequency (number of spots) and total urine volume (μl) at baseline (day 0) and after 10 days of WAS (**A**,**B**) or normal housing (**C**,**D**) in female mice. Data represented as mean ± SEM (n = 7) and analysed by paired Students t test (**p < 0.01 vs baseline).

There were no differences in feeding behaviours between the control and stressed animals and the body weight of mice from both groups were between 18 and 23 g at the time of euthanasia. In stressed animals, urinary frequency and total voided volume were measured to confirm development of bladder overactivity. After 10 days of WAS the frequency of voids was approximately doubled (p < 0.01) compared to baseline (Fig. 1A), while the total volume of voided urine was not significantly different to baseline (Fig. 1B). This is consistent with OAB symptoms of increased frequency without an increase in urine production. Urinary frequency and volume were unchanged in unstressed mice over the 10-day experimental period (Fig. 1C,D).

### Effect of WAS on bladder function and afferent nerve activity

**Figure 2:**
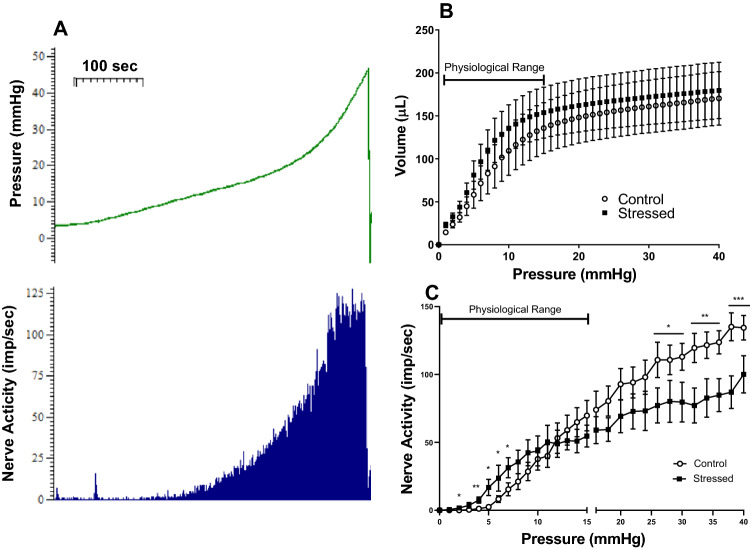
Example pressure and afferent nerve activity trace during bladder distension to 40 mmHg (**A**) in a control bladder. Bladder compliance during filling to 40 mmHg intravesical pressure (**B**) and total afferent nerve activity (**C**) during filling to 40 mmHg, data represented as mean ± SEM (n ≥ 5) and analysed by un-paired Students t test (*p < 0.05, **p < 0.01 and ***p < 0.01 control vs stressed bladders).

Bladders were filled at a rate of 30 μl/min and bladder pressure and nerve activity were recorded. As the volume in the bladder increased both the pressure and nerve activity increased (Fig. 2A). The micturition threshold in mice occurs when intravesical pressure reaches approximately 15 mmHg^24^. Ramp distensions up to 40 mmHg were performed which includes the physiological pressures 0–15 mmHg and continues beyond into the supraphysiological range. Bladder compliance was unchanged by stress during filling at all pressures up to 40 mmHg as seen by the volume-pressure relationship (Fig. 2B) with maximum bladder volumes similar between control (170.3 ± 31.2 µl) and stressed bladders (179.6 ± 32.8 µl). At lower physiological pressures of 4–7 mmHg the nerve activity was significantly higher in stressed bladders than controls (Fig. 2C). For example, at 5 mmHg the afferent activity in stressed bladders was 17 ± 6.0 imp/s compared to 2.4 ± 0.6 imp/s in controls (p < 0.05).

Whereas, at higher noxious pressures above 25 mmHg, the nerve activity was depressed after stress. At maximum pressure, nerve activity was 90.2 ± 11.1 imp/s in stressed bladders and 134.4 ± 9.0 imp/s in controls (p < 0.01). Comparison of mean area under the curve revealed significant differences between the nerve activity responses in the control and stressed group in the physiological and supraphysiological pressure ranges, with a significant increase detected in the stressed group within the physiological range (control 367 ± 48 imp mmHg/s vs stressed 436 ± 51 imp mmHg/s; p < 0.001), while there was a significant decrease with stress in the high-pressure range (control 2608 ± 143 imp mmHg/s vs stressed 1836 ± 155 imp mmHg/s; p < 0.001).

**Figure 3:**
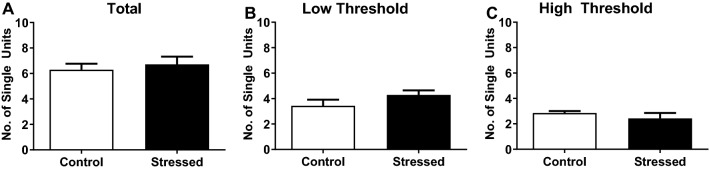
Number of distinct nerve fibres contributing to the total nerve response in each control or stressed bladder (**A**) and how many of these were low threshold (**B**) and high threshold (**C**) nerve fibres in each treatment group. Data represented as mean ± SEM (n = 7) and analysed by un-paired Students t test.

The nerve bundle from the bladder is made up of many fine branches and only one of these branches was inserted into the suction electrode to measure bladder nerve activity. The single branch contains numerous distinct nerve fibres which contribute to the total nerve activity measured and these distinct fibres were identified using Spike2 software wavemark analysis. The number of distinct nerve fibres contributing to the total nerve response from each bladder was not significantly different between treatment groups (Fig. 3A). Each individual fibre was then classified based on the pressure threshold at which it became active. The low threshold nerves start firing at low bladder pressures shortly after bladder filling begins, whereas the high threshold nerves become active at higher pressures around 15 mmHg. Both stress and control bladders had a similar number of fibres start responding at either low pressure thresholds or high pressure thresholds (Fig. 3B,C respectively). The percentage of the total active nerve fibres that had low thresholds was similar between control and stressed bladders (56% ± 4% and 65% ± 5% respectively) indicating that the ratio of low to high threshold fibres contributing to the overall nerve response was unchanged after stress.

**Figure 4:**
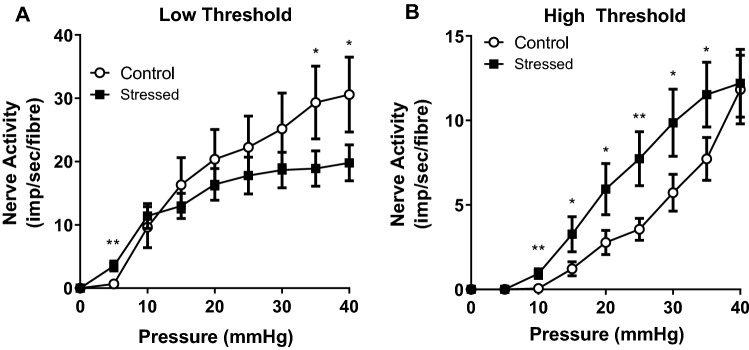
Nerve activity of low and high threshold fibres in control and stressed bladders during bladder filling to 40 mmHg intravesical pressure. Data represented as mean ± SEM (n ≥ 15) and analysed by un-paired Students t test (*p < 0.05 and **p < 0.01 control vs stressed bladders).

The activity of each low or high threshold fibre was then measured. The low threshold nerves, responsible for filling sensations, were significantly more active in stressed bladders (3.5 ± 0.8 imp/s/fibre) than controls (0.7 ± 0.3 imp/s/fibre) at the physiological pressure of 5 mmHg (p < 0.01) (Fig. 4A). At the supraphysiological pressure points of 35 and 40 mmHg the low threshold fibres in stressed bladders were significantly less active than controls (p < 0.05) [19.8 ± 2.8 imp/s/fibre and 30.6 ± 5.9 imp/s/fibre at 40 mmHg in stressed and control groups respectively]. The high threshold fibres, responsible for pain sensations, were more active in the stressed bladders than in controls across all pressure points from 10 to 35 mmHg (p < 0.05) (Fig. 4B) [3.3 ± 1.0 imp/s/fibre and 1.2 ± 0.4 imp/s/fibre at 15 mmHg in stressed and control groups respectively].

### The effect of capsaicin on afferent nerve activity in WAS induced OAB

**Figure 5:**
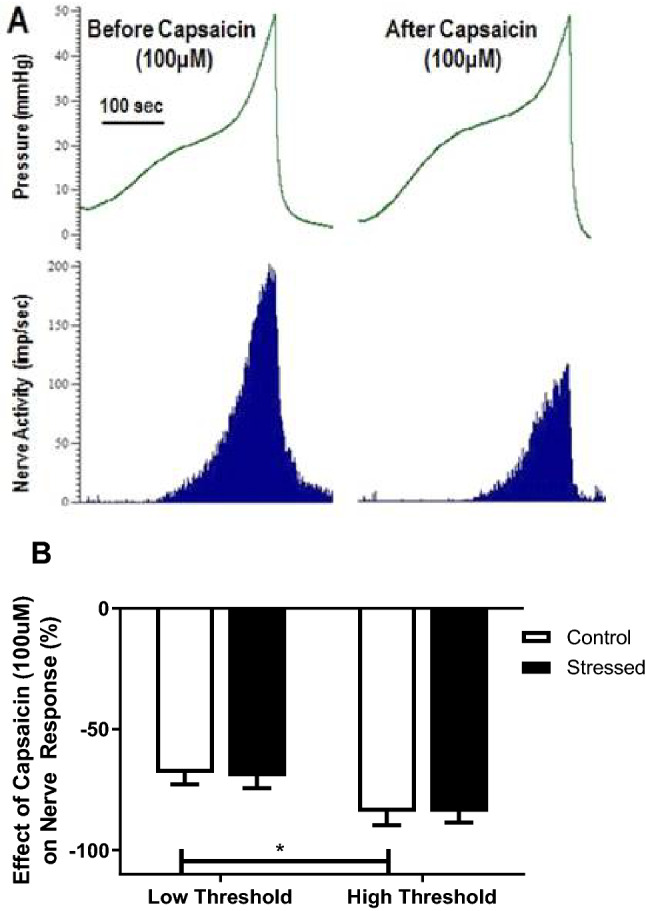
Example pressure (mmHg) and nerve activity (imp/s) traces (**A**) during bladder distension before and after capsaicin (100 μM) in a control bladder. Effect of capsaicin (100 µM) on low and high threshold fibre responses at maximum bladder distension in control and stressed bladders (**B**), data represented as a percentage of the maximum nerve response to distension before capsaicin administration (mean ± SEM, n ≥ 17). Data analysed by Students t test (*p < 0.05 low vs high threshold nerves).

Capsaicin (10 µM and 100 µM) was applied serosally to empty bladders, but the direct, non-distension induced afferent nerve firing response was inconsistent. However, capsaicin (100 µM) consistently reduced the nerve activity in response to bladder distension (Fig. 5A) and this effect was similar in control and stressed groups (Table 1). The individual nerve fibres all demonstrated reduced activity after capsaicin (100 µM), but the reduction varied from total abolition of response to a 20% reduction in response at maximum pressure. The total nerve activity at maximum pressure was reduced by 63.2% ± 5.6% and 65.1% ± 8.1% in control and stressed bladders respectively. Capsaicin appeared to affect the high threshold nerves more than the low threshold nerves, but this was only statistically significant in the tissue from control bladders (p < 0.05) (see Fig. 5B). Stress did not change the effect of capsaicin (100 µM) on the low or high threshold nerves with the reduction in response to distension similar in both groups (Fig. 5B).

**Table 1:** Maximum nerve activity recorded in bladders from control and stressed mice during distension to 40 mmHg before and after application of Capsaicin (100 µM).

	Control (imp/s)	Stressed (imp/s)
Before capsaicin	114.8 ± 12.5	79.4 ± 20.3
After capsaicin	53 ± 12.9	29.1 ± 9.6

Mean ± SEM from n ≥ 5.

### Responses to purinergic and muscarinic receptor stimulation after WAS

**Figure 6:**
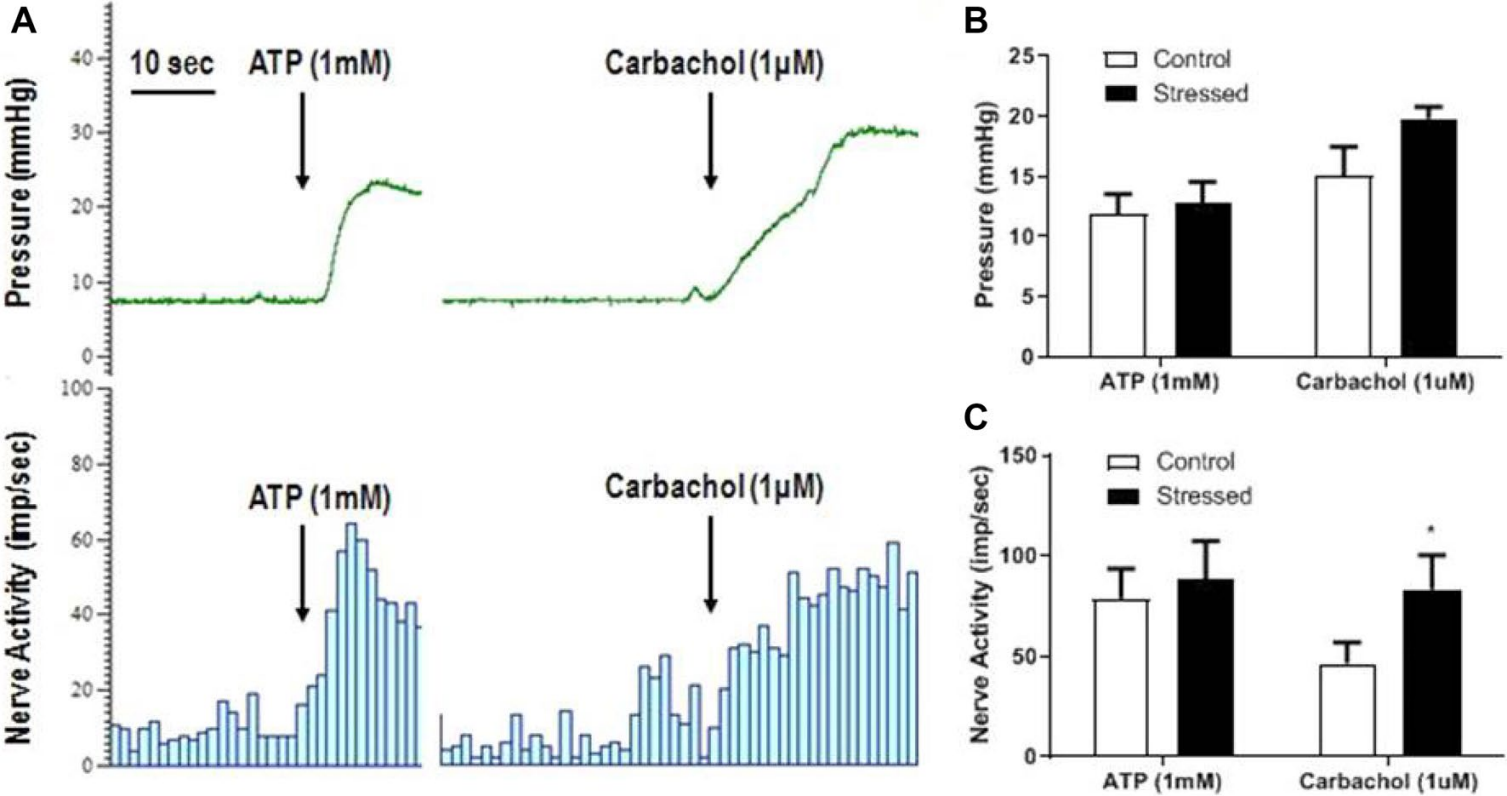
Typical traces showing bladder pressure (mmHg) and nerve activity (imp/s) in response to ATP (1 mM) and carbachol (1 μM) during bladder accommodation (**A**) in a control bladder. Changes in bladder pressure (**B**) and nerve activity (**C**) in control and stressed bladders in response to ATP (1 mM) and carbachol (1 µM), data represented as mean ± SEM, n ≥ 6 and analysed by Students t test (p < 0.05 vs control).

The bladders were filled to 15 mmHg and left to accommodate the volume in the bladder. After pressure and nerve activity had stabilised, the effect of ATP (1 mM) and carbachol (1 μM) could be measured (see Fig. 6A). ATP (1 mM) was applied serosally and caused an increase in bladder pressure (Fig. 6B) and nerve activity (Fig. 6C) and these effects were similar in tissues from control and stressed bladders. After washout, carbachol (1 µM) was then applied serosally and also caused an increase in bladder pressure and nerve activity. The carbachol induced bladder contractions were not significantly different between the control and stressed groups (Fig. 6B), however the associated nerve activity was almost doubled from 46.6 ± 10.2 imp/s in control bladders to 83.1 ± 17.2 imp/s in stressed bladders (p < 0.05, Fig. 6C).

### Effect of WAS on phasic responses of the bladder

**Figure 7:**
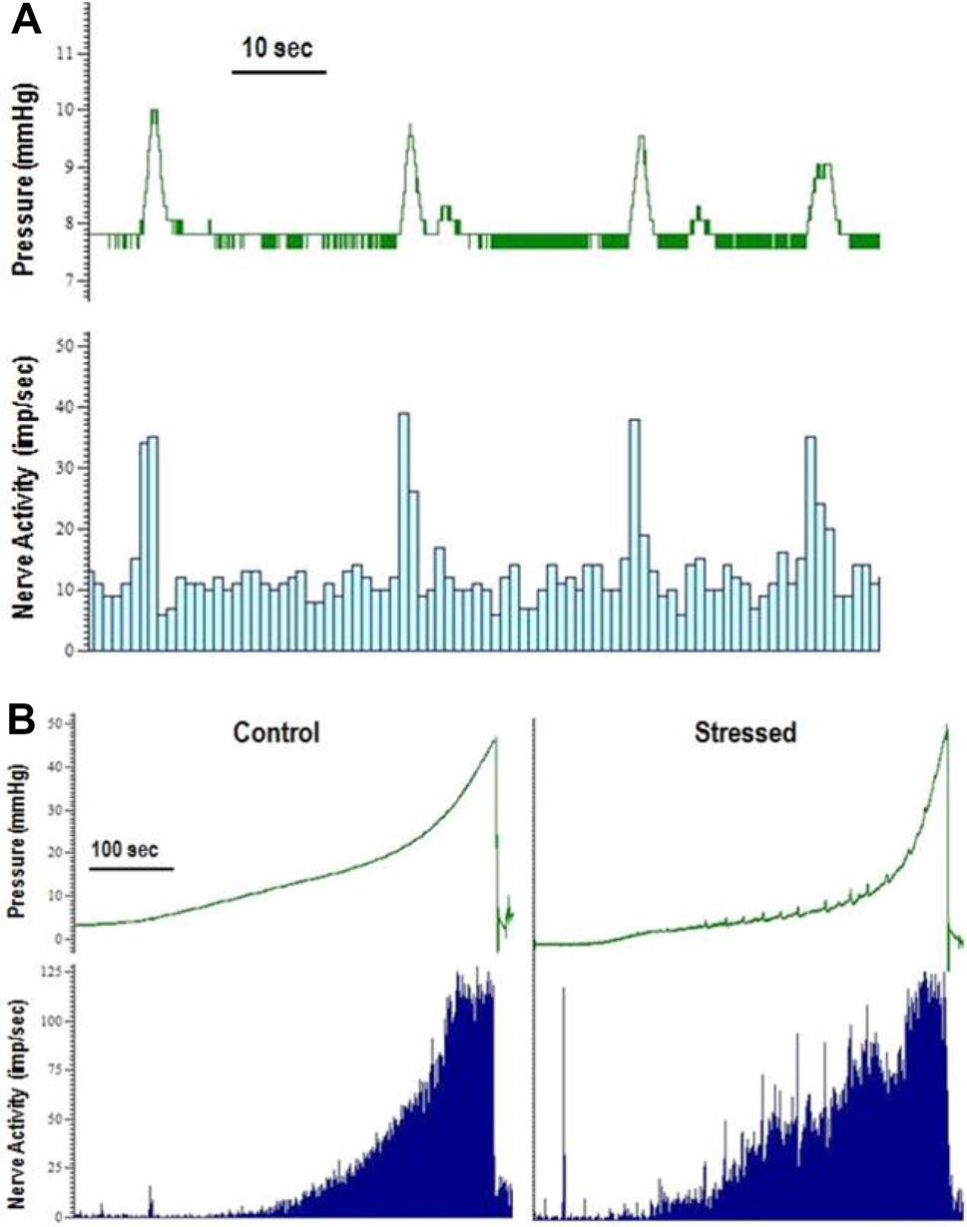
Typical pressure (mmHg) and nerve activity (imp/s) traces showing (**A**) the increase in nerve activity associated with the increase in pressure of phasic contractions in a stressed bladder during accommodation and (**B**) the phasic contractile activity of bladders during distension and the associated nerve activity from a control bladder (no phasic contractions) and a stressed bladder (presence of phasic contractions).

Phasic contractions of the bladder were observed during filling and accommodation phases and these contractions were associated with an increase in bladder afferent nerve activity (Fig. 7A). Phasic contractions were measured during the initial baseline distension, at the end of the accommodation period and during distensions performed 1 h after exposure to carbachol and 10 min after exposure to capsaicin (100 μM). The effect of stress on the frequency and amplitude of phasic contractions as well as the associated nerve activity was measured. Bladders from stressed animals generally demonstrated more phasic activity than control bladders (Fig. 7B). In control bladders only 1 out of 6 showed any phasic activity and that occurred only during bladder distension 1 h after exposure to carbachol (1 µM) (Table 2). Whereas 5 out of 7 of the stressed bladders demonstrated phasic activity under the same conditions.

**Table 2:** Percentage of control and stressed bladders exhibiting phasic contractions of the bladder during various conditions.

Condition	Control (%)	Stressed (%)
Baseline distension	0	57
End of accommodation phase	0	43
Distension after carbachol (1 µM)	17	71
Distension after capsaicin (100 µM)	0	57

The phasic activity exhibited by the stressed bladders was further investigated to determine whether carbachol (1 μM) or capsaicin (100 μM) affected the frequency or amplitude of contractions or the associated nerve activity when compared to those in the initial baseline distensions. Neither carbachol nor capsaicin significantly affected the phasic contractions in the stressed tissues. Although capsaicin appeared to reduce the nerve activity associated with the contractions it was not statistically significant (data not shown).

### The effect of WAS on intravesical mediator release during distension

**Figure 8:**
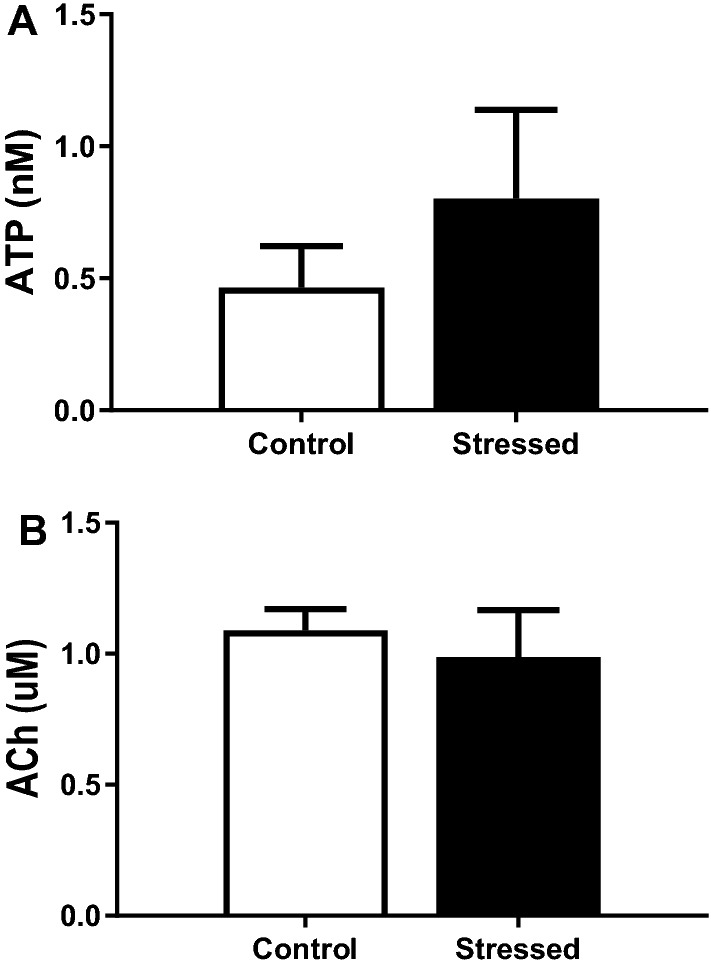
Concentration of ATP (**A**) and ACh (**B**) in intravesical contents collected during distension of control and stressed bladders. Data represented as mean ± SEM, n ≥ 6.

Three bladder distensions to 40 mmHg were performed at the beginning of each experiment and the intravesical contents of the bladder were collected for measurement of ATP and ACh content. Stress did not significantly change the intravesical concentrations of ATP or ACh (Fig. 8A,B, respectively).

## Discussion

The present study confirms that WAS induces bladder overactivity in mice characterised by an increased void frequency without affecting urine production. Bladder compliance in response to distension was similar between stressed and control bladders.

Coordination of bladder function is mediated by multiple reflex pathways initiated by afferent input^25^. Bladder sensations are conveyed primarily by the pelvic nerve but also by the hypogastric nerve^26^. Sensory nerves have been found in the detrusor and suburothelially and respond to stretch of the bladder wall^27^ with urothelial afferents also able to respond to chemical stimuli from within the bladder or urothelium^28^. Afferent projections to the brain terminate in the periaqueductal gray (PAG) which conveys information to the pontine micturition centre (PMC)^29^. When bladder afferent activity exceeds the micturition threshold in the PAG it will activate the PMC and cause voiding. The PAG controls the conscious aspect of voiding by coordinating information to and from higher brain centres to register bladder filling or pain sensations and to delay activation of the PMC^30^.

Compared to controls, stressed bladders demonstrated increased afferent nerve activity at low bladder pressures (4–7 mmHg). The micturition threshold in mice occurs when intravesical pressure reaches approximately 15 mmHg^24^. Therefore, the effects of WAS on afferent activity are relevant to typical physiological filling. This result suggests that stressed animals may experience sensations of bladder filling at lower bladder volumes. Furthermore, it is possible that increased afferent activity could surpass the micturition threshold of the PAG at lower bladder pressures causing the smaller more frequent voids seen in stressed animals. At intravesical pressures above 15 mmHg the nerve activity in stressed bladders was lower than controls although this was only significant at pressures above 25 mmHg. It is not clear why this may have occurred but could be indicative of a compensatory mechanism in response to stress.

Afferent nerves can be classified as low or high threshold according to whether they become active at intravesical pressures below or above 15 mmHg^28,31,32^. In certain conditions previously non-responsive (silent) fibres can become active or high threshold fibres can become low threshold fibres contributing to greater overall nerve activity travelling to the PAG. In the present study the total number of distinct nerve fibres contributing to the overall response was unchanged after stress indicating that the increased nerve activity is due to enhanced firing of individual nerve fibres rather than by activation of silent fibres. Furthermore, the ratio of low and high threshold nerves was similar between groups and therefore it appears that stress did not change the activation threshold of bladder afferents.

At physiological intravesical pressures the low threshold nerves were significantly more active in bladders from stressed animals than in controls. Mingin et al.^16^ also reported increased afferent activity at low bladder pressures up to 10 mmHg after social stress in 6-week old male mice. Interestingly, at pressures above 10 mmHg the activity of the low threshold nerves started to decrease (significant at 35–40 mmHg). The activity of the high threshold nerves was significantly greater in bladders from stressed animals than in controls at all pressure points from 10 to 40 mmHg. It is thought that low threshold nerves contribute to normal micturition reflexes and high threshold nerves to painful sensations. Therefore, the enhanced activity in the low threshold nerves at physiological bladder pressures provides further evidence that stress induced overactivity is associated with increased sensations of bladder filling at lower bladder volumes. Whereas the increased activity of high threshold nerves suggests that stressed animals may also experience increased pain compared to controls. It should be noted that, although voiding behaviour was performed in conscious animals, the model used to record afferent nerve activity was isolated from the effects of spinal reflexes and central nervous system control and therefore the findings are indicative of what may be occurring in vivo.

Bladder afferent fibres can also be classified as either larger myelinated Aδ fibres or smaller unmyelinated C-fibres^28^. In rodents both Aδ fibres and C-fibres contribute to normal micturition sensations^33^ and both types of nerves can have low or high thresholds^28,34^. However, it is thought that C-fibre afferents become sensitised after stress^15,16^. One way to differentiate between these two subgroups is by their sensitivity to capsaicin as the majority of C-fibres but very few A-δ fibres are capsaicin sensitive^35^.

Capsaicin acts via transient receptor potential vanilloid type 1 (TRPV_1_) on bladder afferents to cause activation and subsequent desensitisation^36^. TRPV_1_ channels have been implicated in overactive bladder^37,38^ and psychological stress has been shown to cause a TRPV_1_ associated hyperactivity in the colon^39^. In this study, capsaicin desensitisation of afferent activity was similar between stressed and control bladders. Furthermore, stress did not change the effect of capsaicin on the low or high threshold nerves with the reduction in response similar in both groups. Therefore, water avoidance stress does not appear to cause changes to TRPV_1_ activity in adult female mice.

Phasic contractions have many potential roles in the bladder including maintaining baseline muscle tone, maintaining folding of the urothelium and generating afferent input during filling^40^. Previous work by West et al.^23^ found that bladders from both control and stressed mice exhibited phasic contractions during accommodation of submaximal isolated bladder filling and that the frequency and amplitude of these contractions was unchanged after stress. Similarly, Lee et al.^41^ found that non-voiding contractions during cystometry in rats was unaffected by 10-days of WAS. In the present study only some of the stressed bladders and none of the control bladders demonstrated phasic contractions during initial distensions or accommodation of a submaximal volume and therefore it is not clear whether there was any difference in the frequency or amplitude of phasic contractions after stress. Bladders from stressed animals appeared more likely to develop phasic contractions and it is possible that this phasic tendency may contribute to the stress induced bladder overactivity by amplifying afferent signals or causing activity to reach the micturition threshold earlier.

West et al.^23^ also demonstrated that carbachol (1 µM) initiated phasic contractions in isolated mouse bladders and that 10-day WAS enhanced their frequency and amplitude. A similar response was seen in the present study in that phasic contractions appeared during distensions after application of carbachol (1 µM) and this was more likely to occur in stressed bladders. Unfortunately, the limited spontaneous contractions in control groups prevented a statistical analysis of the afferent activity associated with phasic contractions and whether stress affected this activity.

The bladder muscle responses to serosal application of carbachol or ATP (i.e., intravesical pressure responses) were unaffected by stress. Whereas the afferent nerve activity in the stressed bladders was significantly higher in response to carbachol, but not ATP. Previous work in mice has shown that muscarinic receptor stimulation has a direct inhibitory effect on afferent nerve activity^42^, but it also causes increased detrusor contraction. Therefore, it is possible that the enhanced activity seen in the present study is due to increased afferent sensitivity to bladder pressure increases rather than a direct muscarinic mechanism.

The urothelium can release various mediators in response to stretch including ATP and ACh which can alter bladder sensory nerve activity^43^. Stress did not affect the release of ATP or ACh from the urothelium during distension suggesting these urothelial mediators are not involved in the stress induced afferent hypersensitivity.

The physiological effects of psychological stress are mediated in a number of ways, including via increased epinephrine release from the adrenal medulla and activation of the hypothalamic–pituitary–adrenal axis resulting in the release of corticotropin-releasing factor, urocortins, adrenocorticotropic hormone and ultimately cortisol (corticosterone in rodents)^44^. These mediators of the stress response can have a variety of effects on visceral sensation including in the bladder. A study in mice investigated the effects of epinephrine and corticosterone on excitability of sensory dorsal root ganglia neurones and found they caused hyperexcitability of the neurones due to enhanced voltage-gated Na^+^ currents and suppressed K^+^ currents^45^. Since Na^+^ channels are responsible for the initiation of an action potential and K^+^ channels are responsible for generating the resting membrane potential and regulating repetitive firing^46–48^ it is possible that similar stress induced alterations in these channels may have contributed to the increased afferent activity seen in the present study.

Previous work has shown that WAS can significantly increase urinary frequency in as little as 3 days^23^ and therefore it is possible that the afferent changes observed in the present study may be occurring earlier than the 10-day timepoint investigated. We did not look at post-stress recovery in this study and it is unknown if the changes in bladder afferent nerve activity persist following cessation of stress exposure. To our knowledge only there is only one published study that investigated post-stress recovery, reporting that bladder dysfunction lasts 1 month after stress, however they did not present data in the paper to support this claim^49^. If bladder dysfunction does persist, it suggests that sensory changes may also persist after stress exposure ends. In other systems, duration of stress-induced dysfunction is organ dependent, with full recovery observed in the uterus but irreversible changes in fallopian tubes of rats following 4–12 weeks stress exposure^50^.

In conclusion, water avoidance stress in adult female mice is associated with increased afferent activity at physiological bladder pressures. Both low and high threshold nerves demonstrated increased afferent activity suggesting that stressed animals may experience enhanced bladder filling sensations as well as bladder pain sensations. The number of single nerve fibres and the ratio of low/high threshold fibres contributing to the nerve responses was similar between bladders from stressed and control animals indicating that stress did not activate silent fibres nor did it change the activation threshold of the nerves. Therefore, water avoidance stress appears to increase the inherent excitability of the individual nerve fibres leading to bladder overactivity.

## Methods

### Water avoidance stress model

The study is reported in accordance with ARRIVE guidelines (https://arriveguidelines.org). All procedures were performed in accordance with the Australian Code for the Care and Use of Animals for Scientific Purposes and with the approval of the University of Queensland Animal Ethics Committee (BOND/536/17). Adult female C57BL/6J (12–14 weeks in age; n = 7 in each group) were used in this study and housed under environmentally controlled conditions (23 °C, 60% humidity), with 12-h light–dark cycles and free access to food and water. Mice were randomly allocated into either Control or Water Avoidance Stress (WAS) groups.

WAS is commonly used in rodents to induce a stress response and the protocol used in this study is as previously described^51^. Mice in the WAS group were placed individually on a central pedestal surrounded by water for 1 h/day for 10 consecutive days. Following each stress exposure mice were returned to their normal housing. The control group consisted of age-matched control mice housed under normal conditions and were not exposed to water avoidance stress protocols.

### Voiding pattern analysis

Voiding pattern analysis (VPA) was performed to confirm development of bladder overactivity in the stressed animals. VPA was performed prior to and on completion of the WAS treatment period according to the collection procedure described by West et al.^52^. Briefly, standard mouse cages were lined with ‘Filtech’ hardened ashless filter paper, Quantitative 2 μm grade 225 to absorb voided urine. The mice were placed individually into the lined cage for 2 h at the beginning of the light cycle, with access to food and drinking water. VPA filter papers were collected and urine spots detected using a Molecular Imager ChemiDoc XRS ultraviolet transilluminator (#720BR1293 BioRad, California USA). The papers were photographed, digitized, and then analyzed using ImageJ software (Version IJ. 1.46r; https://prismj.nih.gov/ij/index.html), to measure urine spot size (surface area) and number of voids. A standard curve was constructed using known volumes of urine to allow for conversion of urine spot size to volume.

### Afferent nerve recording

An afferent nerve recording preparation was used for functional bladder studies as previously described^31,32^. Mice were euthanised by cervical dislocation 24 h following final water avoidance stress exposure. The pelvic region was dissected and placed into a 30 mL bath, continually superfused with gassed (95%O_2_/5% CO_2_) Krebs-bicarbonate solution (composition in mM: NaCl 118, NaHCO_3_ 24.9, CaCl_2_ 1.9, MgSO_4_ 1.15, KCl 4.7, KH_2_PO_4_ 1.15, and D-glucose 11.7) at a rate of 6 mL/min at a temperature of 36 °C.

A urethral catheter allowed ramp bladder distension with isotonic saline at 30 μl/min to a maximum intravesical pressure of 40 mmHg. A two-way catheter inserted into the bladder dome allowed measurement of the intravesical pressure and emptying. The ureters were ligated to prevent leakage from the bladder.

The bladder afferent nerve bundle, consisting of a mixed population of pelvic and hypogastric nerves, was divided into fine branches and one branch was cut between the bladder and the spinal cord to allow the free end to be inserted into a suction electrode. A ramp distension to 40 mmHg was performed to confirm that the selected branch was responsive to bladder distension. If the branch did not respond to distension it was deemed to be either a urethral or motor nerve and another branch selected. Multi-unit afferent nerve activity was then recorded by a Neurolog headstage (NL100, Digitimer Ltd, UK), amplified (NL014, Digitimer Ltd), filtered (NL125, band pass filter, Digitimer Ltd) and passed through a 50/60 Hz electrical noise eliminator (Humbug, Quest Scientific, Canada) to a Micro1401 analogue to digital interface (Cambridge Electronic Design, UK) and then visualised on a computer using Spike2 software (Version 7.1, Cambridge Electronic Design, UK; https://ced.co.uk/products/spkovin).

After 30 min equilibration, three bladder distensions were performed to ensure consistent pressure and nerve responses. Bladder accommodation was performed by filling at the same rate to 15 mmHg and allowing the pressure and nerve activity to plateau. From this plateau the muscle and nerve responses to ATP (1 mM) and carbachol (1 µM) were also measured. There was no evidence of additional fibres becoming active after ATP or carbachol instillation that were not active during distension, as might occur if the drugs were stimulating motor fibres, supporting that this method is recording only afferent activity.

A further bladder distension to 40 mmHg was followed by serosal application of capsaicin (10 µM) for 10 min and then an additional application of capsaicin (100 µM). After 10 min exposure another distension was performed and the effect of capsaicin on bladder compliance and afferent nerve activity was determined. The reduction in afferent nerve activity was represented as a percentage of the response recorded before capsaicin application.

### Intravesical mediators and phasic contractions

The intravesical contents from the three baseline distensions were collected and frozen at − 80 °C for later analysis of ATP and ACh content using commercially available assay kits (Molecular Probes, Cat #A22066 and #A12217) and analysed according to manufacturer instructions.

During bladder distension and accommodation phasic intravesical pressure increases were observed and were associated with phasic increases in afferent nerve activity. The frequency (per minute) and amplitude (mmHg) of spontaneous contractions during filling were measured during the 200 s preceding intravesical pressure reaching 20 mmHg and during accommodation were measured during the 200 s at the end of the stabilisation period. The increase in afferent activity associated with each contraction was also quantified and the mean represented as nerve impulses per second.

## Materials

All Krebs-bicarbonate salts and drugs used were obtained from Sigma Aldrich.

### Data analysis

Multi-unit nerve activity was quantified using Spike2 software (Version 7.1, Cambridge Electronic Design, UK; https://ced.co.uk/products/spkovin) which counted the number of action potentials that crossed a pre-set threshold. The threshold in each experiment was set to a level just above the baseline activity recorded when that particular bladder was empty. The afferent nerve response during bladder distension was calculated by measuring the afferent activity (imp/s) at each intravesical pressure increment up to 40 mmHg.

Single-unit (single nerve fibre) sorting was performed offline using the Spike2 wavemark analysis function which discriminates between the distinct action potential characteristics of individual nerve fibres including waveform, amplitude and duration. The action potentials were analysed and templates were constructed based on their distinct shape in order to identify the individual nerve fibres in each preparation. Afferent activity was sampled at 20,000 Hz such that a 1.8 ms period consisting of approximately 28 data points was used to construct each template. The parameters for a spike to match a template were typically < 10% variability in amplitude and > 60% of data points within the template boundary. Principal component analysis scales and represents the differences between spike shapes in 3 dimensions and was used to ensure that spike templates were different enough to be classified as distinct nerve fibres.

All experiments were randomized, with seven mice per experimental group and each experimental protocol started on a different day. All graphical analyses and statistical analysis were performed using GraphPad Prism Version 8 (GraphPad Software, San Diego, USA; https://www.graphpad.com/). Data is presented as mean ± standard error of the mean (SEM). Data were analysed using paired or un-paired Student’s t test and significance levels were defined as p < 0.05(*). Unpaired Student’s test used to compare mean nerve activity responses at each pressure point, in addition area under the curve (AUC) was calculated within the physiological and supraphysiological pressure ranges for nerve activity responses and compared between the control and stressed groups.

### Ethics approval

The work in this study was performed in accordance with the Australian Code for the Care and Use of Animals for Scientific Purposes and with the approval of the University of Queensland Animal Ethics Committee (BOND/536/17).
